# Estimating the lifetime economic burden of stroke according to the age of onset in South Korea: a cost of illness study

**DOI:** 10.1186/1471-2458-11-646

**Published:** 2011-08-13

**Authors:** Hye-Young Kang, Seung-Ji Lim, Hae Sun Suh, Danny Liew

**Affiliations:** 1 College of Pharmacy, Yonsei Institute of Pharmaceutical Sciences, Yonsei University, Incheon, Republic of Korea; 2 Graduate School of Public Health, Yonsei University, Seoul, Republic of Korea; 3 Department of Medicine (St Vincent's Hospital), The University of Melbourne, Melbourne, Australia

**Keywords:** Cost of illness, Republic of Korea, Stroke

## Abstract

**Background:**

The recently-observed trend towards younger stroke patients in Korea raises economic concerns, including erosion of the workforce. We compared per-person lifetime costs of stroke according to the age of stroke onset from the Korean societal perspective.

**Methods:**

A state-transition Markov model consisted of three health states ('post primary stroke event', 'alive post stroke', and 'dead') was developed to simulate the natural history of stroke. The transition probabilities for fatal and non-fatal recurrent stroke by age and gender and for non-stroke causes of death were derived from the national epidemiologic data of the Korean Health Insurance Review and Assessment Services and data from the Danish Monitoring Trends in Cardiovascular Disease study. We used an incidence-based approach to estimate the long-term costs of stroke. The model captured stroke-related costs including costs within the health sector, patients' out-of-pocket costs outside the health sector, and costs resulting from loss of productivity due to morbidity and premature death using a human capital approach. Average insurance-covered costs occurring within the health sector were estimated from the National Health Insurance claims database. Other costs were estimated based on the national epidemiologic data and literature. All costs are presented in 2008 Korean currency values (Korean won = KRW).

**Results:**

The lifetime costs of stroke were estimated to be: 200.7, 81.9, and 16.4 million Korean won (1,200 KRW is approximately equal to one US dollar) for men who suffered a first stroke at age 45, 55 and 65 years, respectively, and 75.7, 39.2, and 19.3 million KRW for women at the same age. While stroke occurring among Koreans aged 45 to 64 years accounted for only 30% of the total disease incidence, this age group incurred 75% of the total national lifetime costs of stroke.

**Conclusions:**

A higher lifetime burden and increasing incidence of stroke among younger Koreans highlight the need for more effective strategies for the prevention and management of stroke especially for people between 40 and 60 years of ages.

## 1. Background

Korea bears a burden of stroke that is at least similar to that in the western world, highlighted by being the number one cause of death [1-4]. However, unlike many western countries, the gravity of this appears to be often under-appreciated, and risk factors are inadequately controlled in Korea. According to the Korean National Health and Nutrition Examination Survey (KNHANES), only a slight reduction in the prevalence of hypertension among people over 30 years of age has been observed, from 29.1% in 1998 to 27.9% in 2005 [5,6]. Similarly, the prevalence of hyperlipidemia for people in the same age group has hardly changed, from 8.6% in 2001 to 8.2% in 2005. The quantification of the costs related to stroke in Korea could highlight the problem and help decision makers assign greater priority to stroke prevention and management.

Many earlier stroke-costing studies have been limited to the cost measurement of stroke for one or two years following the acute event, instead of estimating the lifetime burden [7-9]. A few studies have estimated the long-term cost of stroke based on an incidence-based approach, but none have stratified results according to the age of onset [10-12]. Since stroke often confers permanent disability and recurrence is common, costs incurred by patients suffering stroke at an earlier age would be greater compared to those of patients for whom stroke occurred later in life.

A recent, nationally-representative survey in Korea found the prevalence of stroke among people in their 40s had increased by 90.91% (from 0.22% in 1998 to 0.42% in 2005) [9]. Within the same period, stroke prevalence also increased by 54.76% (from 1.26% to 1.95%) and 58.76% (from 2.91% to 4.62%) among Koreans aged in their 50s and 60s, respectively. There was only a modest increase, if at all, in stroke prevalence among Koreans in their 70s and older (increase by 22.84% and decrease by 4.86%, respectively) [5,6]. The fact the age distribution of stroke patients in Korea is moving toward younger ages indicates that the future economic burden imposed by stroke on the Korean community is set to increase.

In the present study, we aimed to estimate the years of life lost and lifetime costs associated with stroke in Korea, stratified according to the age of onset. Incidence-based estimates of the costs of new strokes in Korea were also projected. The study results are expected to increase understanding of the magnitude of lifetime economic burden of stroke and to urge public health policy makers to develop effective prevention and management strategies for stroke, especially for the middle-aged population.

## 2. Methods

### 2.1. Overview of the modeled analysis

**Figure 1:**
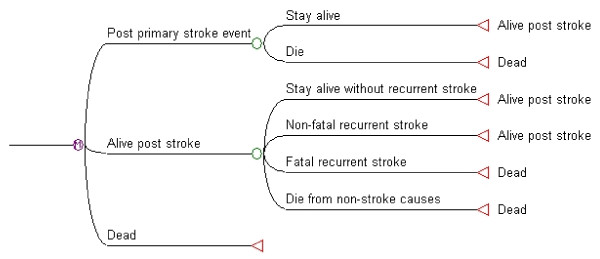
**Markov model to estimate the life-time cost of patients with stroke**.

We developed a state-transition Markov model with yearly cycles to simulate the natural history of stroke among ten gender-and-age specific cohorts of patients [13]. Within each gender, five ages of onset were considered, 45, 55, 65, 75 and 85 years, and follow-up was simulated until the age of 99 years or death. The structure of the Markov model is shown in Figure 1. Three health states were included: 'post primary stroke event', 'alive post stroke' and 'dead.' All subjects in each gender-age cohort began the simulation in the health state 'post primary stroke event', which represented the time at which the primary stroke had just occurred; that is, this was the baseline of the model. In the first year after the primary stroke events, subjects were exposed to two possible transitions: 'stay alive' and 'die'. Transition through the 'die' state was defined by stroke associated with death within 12 months following its diagnosis. The health state 'post primary stroke event' was occupied only during cycle 1 of the model. Survivors moved into the health state 'alive post stroke', from which they could make four possible transitions in subsequent years (cycles): 'stay alive without recurrent stroke,' 'non-fatal recurrent stroke,' 'fatal recurrent stroke,' and 'die from non-stroke causes.' Only transition through either of the first two (non-fatal) transition states led back to the living health state. The health state 'dead' was absorbing.

In each cycle, we calculated the 'expected cost' of stroke multiplying the probability of moving through each transition state by the cost assigned to that state, and then summing these. The sum of the expected costs of all the cycles is therefore the estimated lifetime cost of stroke.

### 2.2. Transition probabilities and data sources

The transition probabilities for fatal (R_fs_) and non-fatal recurrent stroke (R_nfs_) among people with stroke (i.e. transition from 'alive post stroke' to 'fatal recurrent stroke' or to 'non-fatal recurrent stroke') were computed by age and gender as follows:

where p = prevalence of stroke in the general population

R_s _= risk of recurrent stroke (among people with prior stroke)

R_n _= risk of new-onset stroke (among people without prior stroke)

R_t _= risk of acute stroke in the general population

R_nfs _= risk of non-fatal recurrent stroke (among people with prior stroke)

P_nfs _= proportion of non-fatal recurrent stroke (among people with prior stroke)

R_fs _= risk of fatal recurrent stroke (among people with prior stroke)

Gender- and ten-year age-specific epidemiologic parameters for the Korean population (p, R_n_, R_t_, P_nfs_) were derived from a published report provided by the Korean Health Insurance Review and Assessment Services (HIRA) [14]. From the report, stroke was defined according to World Health Organization criteria, including hemorrhagic stroke (international classification of diseases, 10^th ^revision codes I60-I62), ischemic stroke (I63), and non-specific stroke (I64) [15]. In the HIRA report, 'risk of acute stroke event in the general population (R_t_)' and 'risk of acute stroke event among people without stroke (R_n_)' were defined as the attack rate of acute stroke (first-ever + recurrent incidence) per 100,000 population and incidence rate of acute stroke (first-ever) per 100,000 population in 2004, respectively. The distribution of stroke severity, which is usually measured using by neurological scales and functional outcome scales such as Barthel Index, National Institute of Health Stroke Scale, modified Rankin Scale, Glasgow Outcome Scale, and the American Heart Association Stroke Outcome Classification score could not be reflected in the present study because the needed information was not available in the claims data [16,17]. Thus, the severity of stroke was classified as either fatal or non-fatal by according to age and gender.

Transition from 'alive, post stroke' through 'die from non-stroke cause' was defined as the probability of dying due to reasons other than stroke. Due to a lack of necessary data to allow for estimation of non-stroke mortality specifically among Koreans with prior stroke, we applied data from the Danish Monitoring Trends in Cardiovascular Disease (MONICA) study regarding standardized mortality ratios (SMRs) of people post stroke using the following equation [18]:

where, D_s _= non-stroke mortality in the population with prior stroke

D_t _= non-stroke mortality in the general population [19,20]

SMR = standardized mortality ratio of non-stroke mortality among people with prior stroke versus mortality of the general population: 1.914 for males and 2.295 for females

Finally, the transition probability for 'stay alive without recurrent stroke' was simply the complement (the value of it subtracted from one) of the sum of the other two transition probabilities.

Since we used an incidence-based approach to estimate the long-term costs of stroke, the risks mentioned above adhere to the rate concept. Thus, we converted risks (R_fs_, R_nfs_, and D_s_) to probabilities as follows [13]:

where p = probability, r = rate, t = length of cycle

Key parameters of the Markov model are presented in Table 1. As the cycles progressed, relevant input data such as transition probabilities were updated via life-table analysis.

**Table 1:** Key epidemiologic parameters used in the modeled analysis

Age of stroke onset (years)	Prevalence of stroke in general population (%)	Risk of new-onset stroke among people without prior stroke/100,000	**Number of stroke cases (Incidence of stroke in general population/100,000)**^a^	Risk of recurrent stroke among people with prior stroke/100,000	Survival rate of stroke within one year (%)	Risk of non-stroke mortality in general population/100,000
Male						
45~54	0.68	176	6,963 (214)	5,742	82.76	417
55~64	1.95	444	11,615 (577)	7,262	70.42	837
65~74	4.15	1,090	16,493 (1,447)	9,696	65.01	2,128
75~84	6.27	2,022	10,043 (2,777)	14,066	45.27	5,265
≥85	6.96	2,840	2,160 (3,845)	17,286	34.31	17,923
Female						
45~54	0.47	113	4,288 (135)	4,746	82.90	144
55~64	1.56	287	8,100 (376)	6,004	83.36	283
65~74	3.42	758	16,485 (1,053)	9,374	69.66	823
75~84	5.16	1,570	16,639 (2,314)	15,996	49.57	2,982
≥85	4.86	2,471	5,362 (3,118)	15,789	26.35	14,217

^a ^Incidence of stroke in general population with and without prior stroke

### 2.3. Resource use and costs

The model captured all stroke-related resource use and costs, including costs within the health sector, patients' out-of-pocket costs outside the health sector, and costs resulting from loss of productivity due to morbidity and premature death. We presented all costs in 2008 Korean currency value (Korean won = KRW). We did not include disability payments and other forms of government support to estimate stroke-related costs because they do not reflect true societal costs and represent transfers among certain members of society (i.e., transfer costs). In calculating future costs, we did not allow for increases in healthcare costs (the average inflation rate in the 2000s was 2.84% in Korea) nor did we discount future costs to the present value (3% of the discount rate is recommended by Gold et al.), as these opposing effects were similar in magnitude and hence would have nullified one another [21,22].

#### Costs within the health sector

The costs within the health sector consisted of insurance-covered and insurance-noncovered medical costs, informal caregiver costs, and transportation costs to visit health care institutions. Records from the Korean National Health Insurance (NHI) claims database from 2001 to 2004 were used to identify patients with stroke and resource use for insurance-covered medical services associated with stroke. Since the present study was funded by the Korea Ministry of Health, Welfare, and Family Affairs, we were able to obtain the NHI claims data through a formal request process submitted to the Korean National Health Insurance Corporation. Since Korea has a mandatory system for national health insurance, the NHI claims database contains all medical and prescription drug claims records for the entire population. All patients aged 45 or older with at least one claim record of outpatient visits or hospital admissions, containing a primary or secondary diagnosis of stroke (ICD 10 code: I60-64) in 2002 were identified from the NHI claims database. The one-year period prior to 2002 was set to be a 'window period,' such that patients were defined as incident cases only if they didn't have any record of a stroke-related claim during the window period. We defined the case of non-fatal stroke as cases without death within one year after the stroke onset. If patients died within one year after the stroke onset, these cases were defined as fatal stroke. The NHI claims database contained de-identified identification number and we were not able to determine the individual's identity. None of protected health information was included in the received data. All data were stored in a password protected personal computer.

All costs sourced from 2002 to 2004 were inflated to the value of 2008 using annual inflation rates of medical price index for insurance-covered services in Korea [21]. The cost of non-fatal stroke in the first year after its onset was distinguished from the annual costs after the first year. The first-year cost of a non-fatal stroke estimated from the NHI claims database was the average insurance-covered costs in the first year associated with medical and pharmacy costs among stroke patients who did not die within one year after stroke onset. We used the average costs in the second year for a non-fatal stroke case estimated from the NHI claims data as the second-year cost of a non-fatal stroke. It was assumed that beyond the first year after a stroke, annual costs remained constant until recurrence [23]. If a subject experienced a recurrence in a particular Markov cycle that was fatal, then the cost of a fatal stroke was applied to that cycle. The cost of a fatal stroke estimated from the NHI claims database was the average insurance-covered costs associated with medical and pharmacy costs among stroke patients who died within one year after stroke onset. If the recurrence was non-fatal, then first-year cost was applied during that cycle, and subsequent-year costs applied for the subsequent cycles until death or recurrence of stroke. The assumption was made that the costs of a recurrent stroke were the same as costs of a first-ever stroke [24].

Uninsured costs were computed on the basis of a published report for the proportion of uninsured medical costs among the total medical costs spent to treat stroke (29.5% for hospital admission and 15.2% for outpatient visits) [25,26]. Uninsured costs did not include nursing home costs or caregiver costs outside the hospital sector.

We reflected informal caregiver costs by multiplying the average length of stay in the hospital by the average daily wage (56,809 KRW; 1,200 KRW is approximately equal to 1 US dollar) for women aged between 20 and 50 years in 2005, because women in this age group usually take care of their family members who need an informal caregiver [19]. Transportation costs were estimated by multiplying the average round-trip transportation cost to a healthcare institution (891 KRW for outpatient visits and 2,896 KRW for admission) by the total number of stroke-related visits or admissions [5,6]. These published round-trip transportation costs included those of all diseases and were not stroke-specific. Transportation costs for outpatient visits were lower than costs for admission because patients usually visit clinics located nearby for outpatient purposes in Korea, whereas patients who require hospitalization usually visit general hospitals.

Considering elderly patients in Korea generally visit hospitals accompanied by caregivers, transportation costs for a caregiver and informal caregiver costs were summed for patients older than 65 years of age. Informal caregiver costs for outpatient visits were computed as a product of the average number of visits and one-third of the average daily wage, because it was assumed that an outpatient visit takes one-third of a day.

#### Costs outside the health sector

Patients' out-of-pocket spending outside the hospital was derived from a patient's survey undertaken by Lee et al. in 2004. Information collected included costs associated with the utilization of long-term care facilities including nursing homes, medical devices and equipments, commercial caregivers, supplemental drugs, and herbal medicines [27]. The costs of commercial caregivers for stroke victims that are unable to work and who do not stay in a hospital were included in this category. Using the gender- and age-specific ratios of second-year to first-year medical costs (insurance covered and non-covered costs), which ranged from 0.13 to 0.38, out-of-pocket spending for the second year (and beyond) was estimated.

#### Costs resulting from loss of productivity

The costs of loss of productivity due to absence from work and premature death were estimated using a human capital approach only up to the age of 65 years because Korean people generally retire at that age [28]. The costs for absence from work was calculated by multiplying the average daily or hourly wage rates by the number of hospitalization days or hours spent for outpatient visits attributable to stroke. Premature death costs were obtained from the expected value of an individual's future earnings during the potential years of life lost (PYLL), which was defined as life expectancy at the age of death and was obtained from Korean life tables [1,2]. Gender- and ten-year age-specific average yearly wage was used to compute forgone future earnings [19,20]. Loss of productivity due to premature death was applied to patients with fatal strokes and was not applicable to patients with non-fatal strokes.

Productivity costs of absenteeism and premature mortality associated with stroke were also estimated using the friction cost approach as a sensitivity analysis [29,30]. The friction period was assumed to be six months because the unemployment rates in 2008 and 2009 were between 3.2% and 3.6%, which were much lower than the average rate (8.3%) among the countries in the Organisation for Economic Co-operation and Development (OECD) and that in previous literature which used a three-month friction period [29-31]. For training expenditures per employee in Korea, we used the estimate reported by the American Society for Training and Development. Employers in Asia including India, Indonesia, Korea, Malaysia, Pakistan, Philippines, Singapore, Thailand, and Taiwan responded to have invested an average of 362 US dollars per employee on training in 2000 [32]. The training costs were extrapolated from 2000 to a 2008 value using the overall annual consumer price index (129.3 in 2008 based on 2000) [21].

## 3. Results

### 3.1. Years of life lost

From the Markov cohort simulation, the average life expectancy following acute stroke was estimated according to the age of onset. Years of life lost due to stroke were calculated by comparing life expectancy of people with stroke with life expectancy in the age- and gender-matched general population. On average, when stroke (fatal or non-fatal) occurred at the ages of 45, 55, 65, 75, or 85 years for men, the expected years of life lost were 15.57, 14.05, 10.23, 7.40, and 4.46 years, respectively. Overall, women lost more years of life than men after a stroke, with the equivalent age estimates being 17.62, 14.29, 12.61, 9.35, and 5.53 years, respectively. Overall, stroke reduced life expectancy by 48.42 to 86.48% for men and 46.04 to 88.06% for women (Table 2).

**Table 2:** Estimated years of life lost due to stroke

Age of stroke onset (years)	Life expectancy in general population (years)^a^	Estimated life expectancy in patients with stroke^b^	Years of life lost due to stroke^c^	Reduction in life expectancy due to stroke (%)
Male				
45	32.16	16.59	15.57	48.42
55	23.6	9.55	14.05	59.54
65	15.8	5.57	10.23	64.78
75	9.42	2.02	7.40	78.60
85	5.16	0.70	4.46	86.48
Female				
45	38.28	20.66	17.62	46.04
55	28.9	14.61	14.29	49.44
65	19.9	7.29	12.61	63.36
75	11.91	2.56	9.35	78.51
85	6.28	0.75	5.53	88.06

**^a ^**Data source: Korea National Statistical Office, 2005; ^b ^Estimated from Markov cohort simulation; ^c ^Years of life lost due to stroke = (life expectancy in general population - estimated life expectancy in patients with stroke).

### 3.2. Lifetime costs

Annual per-person costs of non-fatal stroke among men aged 45 years or older ranged from 4.8 to 8.4 million KRW during the first year of episode and from 1.3 to 1.9 million KRW in subsequent years, unless there was a recurrence (Table 3). For women, costs ranged from 4.9 to 5.4 million KRW in the first year and 0.6 to 1.7 million KRW in subsequent years (Table 3). For both men and women, first-year costs decreased as patient age increased, whereas subsequent year costs trended in the opposite direction. The detailed cost components (i.e., medical costs, patient's out-of-pocket costs, loss of productivity costs) of the average per-person annual cost associated with non-fatal and fatal stokes are shown in Additional file 1, Table S1 and Additional file 1, Table S2, respectively.

**Table 3:** Average per-person annual costs associated with stroke

Age of stroke onset (years)	Costs of non-fatal stroke (1^st ^year)	Costs of non-fatal stroke (2^nd ^year)	Costs of fatal stroke (including premature death costs)
Male			
45	8,449	1,250	10,543 (521,179)
55	7,324	1,564	11,366 (141,230)
65	5,134	1,530	8,724
75	4,772	1,946	7,490
85	5,075	1,804	6,143
Female			
45	5,358	629	12,182 (171,802)
55	5,032	1,004	9,955 (54,111)
65	4,891	1,309	11,135
75	4,915	1,656	7,993
85	4,669	1,588	5,514

Unit: 1,000 Korean won; All costs are presented in 2008 Korean currency value (1,200 Korean won = 1 US$); Detailed cost components are presented in Additional file 1, Table S1 and Additional file 1, Table S2.

Per-person costs of fatal stroke ranged from 6.1 to 11.4 million KRW for men and from 5.5 to 12.2 million KRW for women. For the age groups starting at 45 and 55 years, the inclusion of loss of productivity costs increased the economic burden of fatal stroke to 521 and 141 million KRW for men, and 172 and 54 million KRW for women, respectively.

According to the Markov cohort simulation, a Korean man was expected to incur lifetime costs of 200.7 million, 81.9 million, 16.4 million, 9.9 million and 6.5 million KRW if he suffered a stroke at the age of 45, 55, 65, 75, or 85 years, respectively (Table 4). The economic impact of stroke was approximately 12.2 or 5.0 times higher, respectively, among people 45 or 55 years old compared with that of 65-year olds. Korean women experiencing stroke at 45, 55, 65, 75, or 85 years of age were likely to incur lifetime costs of 75.7 million, 39.1 million, 19.3 million, 10.8 million, and 6.2 million KRW, respectively (Table 4). Women aged 45 or 55 years had approximately 3.9 or 2.0 times higher lifetime costs compared to women aged 65 years. Male-to-female ratios in lifetime costs were 2.7 to 2.1 among 45 and 55 year olds, respectively, but diminished to near unity after 65 years of age.

**Table 4:** Estimated per-person lifetime costs associated with stroke

Age of stroke onset (years)	Base-case analysis:		Sensitivity analysis	
	Lifetime costs of stroke (excluding premature death costs)	**(1) SMR ± 20%**^a^	**(2) Cost of illness ± 20%**^b^	(3) Friction cost approach
Male				
45	200,701 (36,301)	196,136~205,354	168,978~232,424	47,125
55	81,914 (24,765)	81,748~82,260	67,974~95,854	33,611
65	16,395 (16,395)	15,479~17,505	14,029~18,762	16,395
75	9,888 (9,888)	9,456~10,432	8,138~11,638	9,888
85	6,543 (6,543)	6,314~6,861	5,235~7,852	6,543
Female				
45	75,698 (33,702)	75,454~76,079	64,400~86,997	37,873
55	39,118 (28,052)	38,293~40,086	33,675~44,560	30,948
65	19,261 (19,261)	18,466~20,165	16,558~21,963	19,261
75	10,846 (10,846)	10,449~11,298	8,982~12,709	10,846
85	6,182 (6,182)	6,182~6,182	4,946~7,418	6,182

Unit: 1,000 Korean won; All costs are presented in 2008 Korean currency value (1,200 Korean won = 1 US$); ^a ^SMR: standardized mortality ratio of non-stroke mortality among people with prior stroke versus general population (Base-case values are 1.914 for males and 2.295 for females. Sensitivity analyses were performed using the base case value of SMR ± 20%); ^b ^Cost of illness refers to costs of treating non-fatal stroke (costs in first year and second year), costs of treating fatal stroke, and costs of premature death.

Sensitivity analyses were performed for 20% ± the base-case value of the selected variables: standardized mortality ratio of non-stroke mortality among people with prior stroke versus that of the general population (SMR) and cost variables. We also presented the results of sensitivity analyses using the friction cost approach. The impact of uncertainty of these variables on the estimated lifetime cost of stroke is presented in Table 4.

By applying the estimated incidence rate of stroke in 2004 to the national population for each age and gender stratum of the same year, we projected the total number of incident strokes [1,2,12]. Multiplication of the total number of incident strokes by per-capita lifetime cost of stoke yielded a 4.4 trillion KRW lifetime economic burden attributable to stroke occurring in 2004.

**Figure 2:**
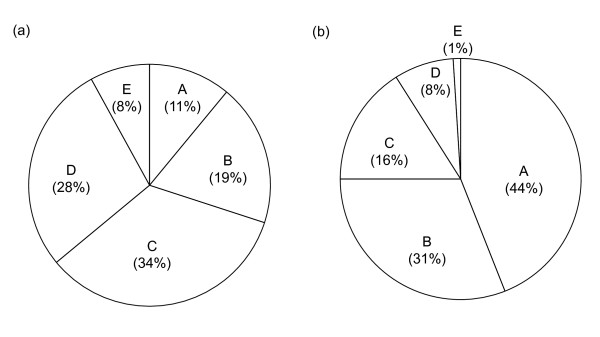
**Age distribution of the incidence (a) and the national economic burden (b) of stroke in Korea**. (A: 45-54 years old, B: 55-64 years old, C: 65-74 years old, D: 75-84 years old, E: 85 years old and above).

Figure 2 presents data on incident stroke and the national economic burden of stroke by various ages. While people in the 45 to 54 and 55 to 64 year age groups accounted for only 11% and 19% of incident strokes, respectively, they accounted for 44% and 31% of the national lifetime costs of stroke. In contrast, Koreans aged 65 years and above contributed to 70% of incident strokes but accounted for only 25% of the national lifetime costs of stroke.

## 4. Discussion

This study is the first to estimate the lifetime costs of stroke in Korea. Previous studies mostly measured the annual cost of stroke using a prevalence-based approach or were limited to the estimations of costs over the short-term (one or several years following the event) [27,33]. Since half of the survivors from stroke are left with permanent disability and consequently bear a lifetime burden, an incidence-based approach has the advantage of providing a more comprehensive picture of the health and economic impact of stroke [34].

The present study showed how the cost of stroke varied according to the age of onset. As commonly observed in earlier studies, per-capita lifetime costs were greater in younger stroke patients mainly due to long-term management and loss-of-productivity costs [12]. In the present study, the expected lifetime cost of stroke for men experiencing acute stroke at the age of 45 years was estimated as 200.7 million KRW, whereas that for men 65 years of age was 16.4 million KRW, a difference greater than 12-fold. Although the difference was less compared to that of men, the lifetime costs for women suffering stroke at 45 years (75.7 million KRW) were still approximately 3.9 times higher than those for women suffering stroke at 65 years (19.3 million KRW).

It has been observed that the average age of new-onset stroke is decreasing in Korea. While the proportion of stroke patients aged in their 70s and 80s has increased by 22.84% and decreased by 4.86%, respectively, between 1998 and 2005, the proportion of patients in their 40s and 50s has increased by 90.91% and 54.76%, respectively [1]. The trend toward younger stroke patients in Korea raises a concern for the national economy because of an erosion of the work force. Additionally, the present study showed that 75% of the national lifetime costs of stroke were attributable to patients aged less than 65 years, although this age-group accounted for only 30% of incident strokes (Figure 2). Consequently, if the average age of stroke patients in Korea continues to decline, then the economic burden of stroke is expected to increase substantially.

Lifetime costs of stroke are greater among men than women due to greater indirect costs (i.e., loss of productivity). The average wage rate in Korea is higher for men than women. Another possible explanation is that the value of women working as housewives and caregivers was not considered, resulting in underestimation of the productivity loss in women. Notably, medical costs were also higher among men than women throughout the different age groups, which is different from data from other countries. For example, according to the summary of published international data between 1993 and 2003 on the costs of stroke, indirect costs were greater for men but direct costs were greater for women [12]. Because it is unknown whether males suffer more severe strokes than females, an unbalanced utilization of healthcare between the genders is possible.

Two methods are available to estimate health-related productivity changes. The human capital approach, which is widely used, is the traditional approach to measure and valuate the potential loss of productivity for all income lost due to absence from work and premature death. This approach is criticized because the true cost imposed to society is usually overestimated [28]. The friction cost approach assumes workers will be replaced after a certain period of time, called the friction period, and the amount of lost productivity depends on the time and cost of replacement. This approach attempts to measure the actual loss of productivity in the economy and usually provides much lower estimates for the loss of productivity than do the estimates from the human capital approach [28,30]. However, we prefer the human capital approach for several reasons. First, the objective of the present study was to evaluate the value of a lost and damaged human life due to stroke rather than estimating the actual monetary loss of productivity in the industry. Second, economic activity returns back to the natural rate of unemployment in the long-run according to the macroeconomic theory. Lastly, there are too many uncertainties to estimate the actual loss of productivity using the friction cost approach because the data about the friction period and employer costs (i.e., hiring and training costs) are difficult to estimate. To obtain the estimates of employer costs needed for the friction cost approach, we searched published literatures and reports intensively to find Korean data, but failed to identify the needed estimates. Government organizations in Korea were also contacted (departments related to human resources and vocational training in the Ministry of Strategy and Finance and Ministry of Employment and Labor) to seek expert opinions on the estimates; however, these searches were unsuccessful. Fortunately, we were able to find the published report of the American Society for Training and Development which contained international comparisons regarding employee training. However, since the report did not include data for Korea only, the estimates for Asia (an average of 362 US dollars per employee on training in 2000) were used, which included nine countries where the number of included Korean companies responding to the survey was not known [32]. As expected, the friction cost approach gave us produced much lower estimates for the loss of productivity (23% and 41% of the expected lifetime costs of stroke estimated by a human capital approach for men experiencing acute stroke at the age of 45 and 55 years, respectively).

One of the major strengths of the present study is that the estimation of the lifetime cost of stroke was based on national data, both in terms of unit costs (i.e., claims records of NHI) and epidemiologic information. This provides greater generalizability of results, compared to that of earlier studies based on patient records from selected hospitals or local stroke registries [10,24]. Another strength of the study was the application of age- and gender-specific cost and epidemiologic data. Survival rate, recurrence rate, and cost of stroke treatment are well known to be affected by patient age and gender [12].

Several assumptions were made in the Markov model that may have led to over or underestimation of stroke costs. First, due to the lack of data, it was assumed that the cost for a recurrent stroke was the same as the cost for the first stroke. However, if the cost of treating a recurrent stroke was more expensive than the first-ever stroke due to the increased severity, then the model would have underestimated the true cost. Secondly, in estimating the costs associated with premature death, we assumed that there was no loss of productivity costs after the retirement age of 65 years. This conservative assumption may also have contributed to the underestimation of the true costs of stroke. On the other hand, the universal application of transportation and loss of productivity costs to all hospital admissions and office visits due to stroke may have led to the overestimation of the true costs of stroke. Additionally, the choice of a human capital approach instead of a friction cost approach in estimating loss of productivity cost would cause overestimation of the costs.

The present study has several limitations. First, although the study has high external validity because of the use of NHI claims data from the entire population in Korea, case ascertainment for stroke solely based on diagnostic codes of insurance claims records may be less accurate compared to a community-based incidence cohort study [10,24]. The validity issue of administrative database coding for stroke cannot be easily resolved since the database is constructed for reimbursement purposes and not for clinical purposes. However, the NHI claims data has a higher reliability than any other data in Korea to estimate the healthcare utilization and costs in terms of representativeness and generalizability since the NHI claims data include the entire Korean population. Moreover, the accuracy of diagnosis codes tended to be higher for claims of severe conditions such as stroke compared with those of other mild conditions [35,36]. In addition, all claims data in Korea is submitted electronically, thus the precision of data is higher than that of the paper-based claims data. Second, due to the lack of detailed epidemiologic data, we applied the same transition probability of having a recurrent stroke regardless of how many cycles or years passed since the initial stroke. Third, information on patients' out-of-pocket expense outside the hospital may have limited generalizability since it was derived from patient survey results from a selected hospital. Fourth, we were not able to incorporate the distribution of severity of stroke measured by neurological scales and functional outcome scales into the analysis because these clinical values were not available in the claims database. Ignoring the distribution of stroke severity may cause distortions in the estimation results of stroke impact.

Last, we did not reflect the value of productivity loss of housewives with stroke. Moreover, we did not consider the productivity loss of family caregivers, who are usually women who do not work outside the home and take care of the stroke victims that are unable to work and do not stay in hospital. Accordingly, the value of stroke in Korea may have been underestimated.

Further research is needed to examine whether the true costs of treatment for stroke patients are different between the results obtained from the claims database and from the patient registry data which include clinical variables representing the severity of stroke.

## 5. Conclusions

In conclusion, the projected per capita lifetime cost among patients with stroke substantially varied depending on the age of onset. Although only 30% of incident stroke occurs in patients aged 45 to 64 years, this age-group accounts for over 75% of the national lifetime costs of stroke. This result confirms the recent trend toward younger stroke patients in Korea and presents a real economic concern. If the age distribution of stroke patients in Korea keeps moving toward younger ages, the future economic burden on the Korean community imposed by stroke will be substantial. Therefore, more effective strategies and efforts targeted at preventing stroke in the middle-aged population should be implemented in Korea, in both public health as well as private clinic settings. Hopefully, the present national epidemiological study will increase the awareness of the economic burden of stroke among the younger population and support public health policy makers in the development of effective prevention and management strategies for stroke.

## Competing interests

The authors declare that they have no competing interests.

## Authors' contributions

HYK developed the study design, interpreted the data, and drafted the manuscript. SJL performed the statistical analysis. HSS interpreted the data and results, and prepared the manuscript. DL provided methodological advice and interpreted the results. All authors have read and approved the final manuscript.

## Pre-publication history

The pre-publication history for this paper can be accessed here:

http://www.biomedcentral.com/1471-2458/11/646/prepub

## Supplementary Material

Additional file 1 **Detailed cost components of the average per-person annual costs**. Table S1 and Table S2 show the detailed cost components (i.e., medical costs, patient's out-of-pocket costs, loss of productivity costs) of the average per-person annual cost associated with non-fatal stroke and fatal stroke, respectively, by age and gender. Click here for file
