# Supplementary material for: Estimating the lifetime economic burden of stroke according to the age of onset in South Korea: a cost of illness study
Source: BMC Public Health. 2011 Aug 13;11:646. doi: 10.1186/1471-2458-11-646 (PMC3171726; doi:10.1186/1471-2458-11-646)
Supplement: Additional file 1 — Detailed cost components of the average per-person annual costs. Table S1 and Table S2 show the detailed cost components (i.e., medical costs, patient's out-of-pocket costs, loss of productivity costs) of the average per-person annual cost associated with non-fatal stroke and fatal stroke, respectively, by age and gender. [file 1471-2458-11-646-S1.DOC]

Additional file 1, Table S1. Detailed cost components of the average per-person annual costs associated with non-fatal stroke

| Age of stroke onset (years) | Costs within the health sector | | | | Costs outside the health sector: Patient’s out-of-pocket spending^d^ | Costs resulting from loss of productivity due to morbidity^e^ | | Total |
| --- | --- | --- | --- | --- | --- | --- | --- | --- |
|  | Medical costs  (insured and uninsured)^a^ | Informal care^b^ | | Transportation^c^ |  |  |  |  |
| ***1^st^ year of the stroke*** | | | | | | | | |
| Male | | | | | | | | |
| 45 | 3,033,876 | 1,091,318 | 10,056 | | 1,653,801 | 2,659,702 | | 8,448,753 |
| 55 | 2,762,172 | 1,167,615 | 10,596 | | 1,653,801 | 1,729,890 | | 7,324,073 |
| 65 | 2,390,515 | 1,074,244 | 15,893 | | 1,653,801 | 0 | | 5,134,453 |
| 75 | 2,070,876 | 1,032,986 | 14,163 | | 1,653,801 | 0 | | 4,771,826 |
| 85 | 2,170,691 | 1,236,712 | 13,574 | | 1,653,801 | 0 | | 5,074,778 |
| Female | | | | | | | | |
| 45 | 2,369,914 | 725,946 | 5,168 | | 1,653,801 | 603,481 | | 5,358,310 |
| 55 | 2,180,219 | 824,792 | 6,501 | | 1,653,801 | 366,535 | | 5,031,847 |
| 65 | 2,238,410 | 988,384 | 10,799 | | 1,653,801 | 0 | | 4,891,395 |
| 75 | 2,110,620 | 1,139,716 | 11,214 | | 1,653,801 | 0 | | 4,915,351 |
| 85 | 1,799,246 | 1,197,540 | 18,459 | | 1,653,801 | 0 | | 4,669,046 |
| ***2^nd^ year of the stroke*** | | | | | | | | |
| Male | | | | | | | | |
| 45 | 535,078 | 103,229 | 2,820 | | 291,677 | | 317,302 | 1,250,107 |
| 55 | 687,509 | 185,869 | 3,705 | | 411,634 | | 275,375 | 1,564,092 |
| 65 | 729,485 | 184,019 | 5,987 | | 504,671 | | 0 | 1,530,075 |
| 75 | 795,279 | 238,068 | 5,568 | | 635,110 | | 0 | 1,946,266 |
| 85 | 725,793 | 329,453 | 6,262 | | 552,966 | | 0 | 1,804,090 |
| Female | | | | | | | | |
| 45 | 302,125 | 55,804 | 1,132 | | 210,832 | | 59,513 | 629,405 |
| 55 | 470,396 | 120,832 | 1,913 | | 356,818 | | 53,697 | 1,003,655 |
| 65 | 624,291 | 191,191 | 3,316 | | 461,244 | | 0 | 1,309,465 |
| 75 | 734,966 | 295,762 | 3,812 | | 575,891 | | 0 | 1,655,948 |
| 85 | 602,936 | 368,002 | 6,053 | | 554,197 | | 0 | 1,587,821 |

All costs are presented in 2008 Korean currency value (1,200 Korean won = 1 US$); ^a^ Costs occurred in acute hospitals and pharmacies including insurance covered costs and uninsured costs which patients have to pay out-of-pocket; ^b^ Informal caregiver costs by family members during hospitalization and outpatient visits (only for patients above 65 years old); ^c^ Round-trip transportation costs to healthcare institutions; ^d^ Patient’s out-of-pocket spending outside the hospital associated with the utilization of long-term care facilities including nursing homes, medical devices and equipments, commercial caregivers, supplemental drugs, and herbal medicines. For the second year, it was estimated using the gender- and age-specific ratios of second-year to the first-year medical costs. For example, the cost of 291,677 for 45-year old male was calculated as: 1,653,801 x 535,078/3,033,876; ^e^ Costs resulting from loss of productivity due to morbidity estimated by a human capital approach only until age 65 years.

Additional file 2, Table S2. Detailed cost components of the average per-person costs associated with fatal stroke

| Age of stroke onset (years) | Costs within the health sector | | | Costs outside the health sector: Patient’s out-of-pocket spending^d^ | Costs resulting from loss of productivity | | Total  (including premature death costs) |
| --- | --- | --- | --- | --- | --- | --- | --- |
|  | Medical costs (insured and uninsured)^a^ | Informal care^b^ | Transportation^c^ |  | Due to morbidity^e^ | Due to premature death^f^ |  |
| Male | | | | | | | |
| 45 | 6,103,427 | 1,111,435 | 5,787 | 826,901 | 2,495,701 | 510,636,001 | 10,543,251  (521,179,252) |
|  |  |  |  |  |  |  |  |
| 55 | 6,725,620 | 1,534,417 | 5,408 | 826,901 | 2,273,329 | 129,864,042 | 11,365,675  (141,229,717) |
|  |  |  |  |  |  |  |  |
| 65 | 6,173,087 | 1,715,769 | 8,687 | 826,901 | 0 | 0 | 8,724,444 |
|  |  |  |  |  |  |  |  |
| 75 | 4,985,126 | 1,668,838 | 8,848 | 826,901 | 0 | 0 | 7,489,713 |
|  |  |  |  |  |  |  |  |
| 85 | 3,942,720 | 1,365,274 | 8,557 | 826,901 | 0 | 0 | 6,143,452 |
|  |  |  |  |  |  |  |  |
| Female | | | | | | | |
| 45 | 9,041,724 | 1,323,030 | 4,013 | 826,901 | 986,216 | 159,619,779 | 12,181,884  (171,801,663) |
|  |  |  |  |  |  |  |  |
| 55 | 7,085,190 | 1,410,966 | 5,284 | 826,901 | 627,028 | 44,155,269 | 9,955,370  (54,110,639) |
|  |  |  |  |  |  |  |  |
| 65 | 8,079,026 | 2,219,628 | 9,481 | 826,901 | 0 | 0 | 11,135,036 |
|  |  |  |  |  |  |  |  |
| 75 | 5,301,649 | 1,856,218 | 8,384 | 826,901 | 0 | 0 | 7,993,152 |
|  |  |  |  |  |  |  |  |
| 85 | 3,348,494 | 1,327,622 | 11,122 | 826,901 | 0 | 0 | 5,514,139 |
|  |  |  |  |  |  |  |  |

All costs are presented in 2008 Korean currency value (1,200 Korean won = 1 US$); ^a^ Costs occurred in acute hospitals and pharmacies including insurance covered costs and uninsured costs which patients have to pay out-of-pocket; ^b^ Informal caregiver costs by family members during hospitalization and outpatient visits (only for patients above 65 years old); ^c^ Round-trip transportation costs to healthcare institutions; ^d^ Patient’s out-of-pocket spending outside the hospital associated with the utilization of long-term care facilities including nursing homes, medical devices and equipments, commercial caregivers, supplemental drugs, and herbal medicines; ^e^ Costs resulting from loss of productivity due to morbidity estimated by a human capital approach only until age 65 years; ^f^ Costs resulting from loss of productivity due to premature death estimated by a human capital approach only until age 65 years.
